# Genetics in Diabetic Retinopathy: Current Concepts and New Insights

**DOI:** 10.2174/13892029113149990008

**Published:** 2013-08

**Authors:** Olga Simó-Servat, Cristina Hernández, Rafael Simó

**Affiliations:** 1 Diabetes and Metabolism Research Unit. Vall d’Hebron Research Institute. Universitat Autònoma de Barcelona, Spain;; 2 Centro de Investigación Biomédica en Red de Diabetes y Enfermedades Metabólicas Asociadas (CIBERDEM), ISCIII, Spain

**Keywords:** Diabetic retinopathy, Genetics, Genome-wide association studies, Linkage studies.

## Abstract

There is emerging evidence which indicates the essential role of genetic factors in the development of diabetic retinopathy (DR). In this regard it should be highlighted that genetic factors account for 25-50% of the risk of developing DR. Therefore, the use of genetic analysis to identify those diabetic patients most prone to developing DR might be useful in designing a more individualized treatment. In this regard, there are three main research strategies: candidate gene studies, linkage studies and Genome-Wide Association Studies (GWAS). In the candidate gene approach, several genes encoding proteins closely related to DR development have been analyzed. The linkage studies analyze shared alleles among family members with DR under the assumption that these predispose to a more aggressive development of DR. Finally, Genome-Wide Association Studies (GWAS) are a new tool involving a massive evaluation of single nucleotide polymorphisms (SNP) in large samples. In this review the available information using these three methodologies is critically analyzed. A genetic approach in order to identify new candidates in the pathogenesis of DR would permit us to design more targeted therapeutic strategies in order to decrease this devastating complication of diabetes. Basic researchers, ophthalmologists, diabetologists and geneticists should work together in order to gain new insights into this issue.

## INTRODUCTION

Diabetic retinopathy (DR) is the main cause of blindness in working-age adults in developed countries. It is well known that diabetes duration, poor glycemic control, and hypertension are the primary factors accounting for the risk of developing DR [1-13]. Therefore, the available tools to prevent this disease are a tight control of both blood glucose levels and hypertension, as well as the implementation of the current screening guidelines. Clinical studies on human subjects with diabetes reveal a substantial variation in the onset and severity of retinopathy that is not fully explained by the known risk factors [14-17]. Indeed, in the ADVANCE Trial intensive glucose control to reduce glycosylated hemoglobin to 6.5% or lower had no effect on the 5-year incidence of DR [16]. In the same study, the lowering of blood pressure to near normal levels did not achieve a further reduction in the progression of DR [16]. In addition, some patients with poor control of glycemia or blood pressure do not develop DR [17]. These data suggest that genetic factors play an essential role in accounting for the susceptibility to developing this late diabetic complication.

Genetic factors may influence either the onset or the severity of DR. In fact, heritability estimates ranging from 25% to 50% have been reported for proliferative DR [18, 19]. Also the study of identical twins found a concordance for DR (68% in type 1 diabetes and 95% in type 2) [20] and in the Diabetes Control and Complication Trial cohort, the odds ratio for severe retinopathy when a relative had retinopathy was 3.1 [15]. Similar findings have been replicated in other familial risk studies [21-23].

Genetic susceptibility to retinopathy is also suggested by the findings in some studies of a greater prevalence of retinopathy among certain ethnic groups. In the Veterans Affairs Diabetes Trial, the prevalence of moderate to severe DR was found to be higher for Hispanics and African-Americans than for non-Hispanic whites [24]. However, ethnicity did not explain the difference in the prevalence of DR in another large study [25].

With the study of the genetics of this disease we will be able to identify genome variants that are associated with an increased risk of complications among diabetic subjects, with the expectation that the location and function of these variants will point to genes and hence molecular pathways that are involved in the etiology of DR. Such genetic approach may also generate algorithms for the early identification of diabetic individuals at a high risk of complications who may benefit from especially aggressive prevention programs. In this regard there are three main research strategies, namely; candidate genes studies, linkage studies in families and Genome-Wide Association Studies (GWAS).

## Method of Literature Review

References for this review were identified through a comprehensive literature search of the electronic MEDLINE database (2000-2013) using the PubMed search service. Search terms included: diabetic retinopathy, genetics, genes, Genome-Wide Association Studies, linkage studies. The search was conducted in January-April 2013 and all abstracts were reviewed and relevant articles retrieved. In addition, reference lists from the selected articles were used to obtain further articles not included in the electronic database.

### Candidate Genes Studies

In the candidate gene approach, several genes encoding proteins closely related to DR are analyzed, usually in case-control studies. Most candidate gene studies have examined the genetic variants implicated in diabetes development or metabolic pathways such as the polyol pathway, the formation of advanced glycation end products (AGE) and hypoxia induced angiogenesis through vascular endothelial growth factor (VEGF).

These kinds of studies are usually based on small patient samples, and they often yield inconsistent results. To circumvent this issue, meta-analyses have been undertaken to pinpoint the few genes for which there might be cumulative evidence for an association with DR. 

#### Aldose Reductase Gene

The polyol pathway is a major metabolic pathway linking hyperglycemia to diabetic complications, and aldose reductase (ALR2) is the first and rate limiting enzyme of this pathway. ALR2 converts glucose to sorbitol in an NADPH-dependent reaction. Hyperglycemia leads to intracellular sorbitol accumulation, thus resulting in osmotic stress, which plays an essential role in the development of diabetic retinopathy [26]. 

The gene encoding ALR2 is located on chromosome 7q35, and a meta-analysis has found that the ALR gene (*AKR1B1*) is the most significantly associated with DR [27]. Associations of three ALR2 SNPs (single nucleotide polymorphisms) with DR have been reported in the literature: the promoter SNP rs759853, the (CA)n microsatellite polymorphism located at 5’ of the ALR gene, and more recently, SNP rs9640883. However, it has been suggested that the commonly reported association of AKR1B1 with DR may be due to an association of this polymorphism with a younger age at the onset of diabetes rather than its direct effect on DR development [28].

Three alleles of (CA)n microsatellite have been related to DR. A significant association with z-2 allele and DR (both non-proliferative and proliferative DR) has been found in type 1 and type 2 diabetes. By contrast, the z+2 allele has been reported as protective against the development of DR [27, 29]. In addition, a T allele of the promoter SNP rs759853 also seems to confer protection against DR development. Finally, it has been found that SNP rs9640883 is associated with DR but, as occurs with AKR1B1, this association may reflect the effect that this gene has on the age of onset of diabetes and, therefore, on diabetes duration, rather than a direct effect on increasing the risk of DR [27]. 

#### VEGF

Vascular endothelial growth factor (VEGF), it is an endothelial cell-specific mitogen and has been implicated as a major contributor to the development of DR [30, 31]. Both hypoxia and hyperglycemia stimulate VEGF expression, and in consequence elevated VEGF and its receptor expression have been demonstrated in diabetic retinas [30-33]. 

The *VEGF* gene is located in chromosome 6 (6p21.3). Many SNP’s have been associated with DR, most of them located in the promoter region of the gene [34]. The most important one is the +405 genotype, which has been implicated in a number of diseases, in particular those with an angiogenic basis, like DR. Several studies conducted in the Japanese population have demonstrated an association between the +405 CC genotype or the C allele and the presence of DR [35-37]. However, this association has not been confirmed in Caucasian population [38]. Interestingly, it has been demonstrated that the C allele is associated not only with higher serum levels of VEGF [34] but also with a higher expression of VEGFA in the human retina [39]. In addition, it has also been associated with an increased risk of developing diabetic macular edema [35]. On the other hand, the GG genotype of this polymorphism (compared with the CC genotype) has been found to be an independent predictor of PDR in other studies performed in Caucasian population [40, 41]. Finally, through Indian population, the CG genotype has been associated with DR [42, 43]. Nonetheless, a meta-analysis considering the G allele the risk variant, did not find any statistically significant association between this polymorphism and DR [28]. 

In a Caucasian population, Bleda *et al.* [41] found a relation between the CA genotype of the -2578 polymorphism, which is located in the promoter region of the VEGF gene, and a susceptibility to developing DR. However, this result has not been confirmed in other studies [28, 44]. Also in a Caucasian population, Carter *et al.* [45] found an association between PDR and the SRp55 2994 polymorphism, which is one of the splicing factors that control alternative splicing of the last exon of VEGF pre-RNA, a key element in the balance of pro- and anti-angiogenic VEGF isoforms. 

While individual SNPs showed modest associations with DR, multiple VEGFA variants, assessed by a multi-SNP test, were associated with the development of severe DR in Caucasian type 1 diabetic patients from the Diabetes Control and Complications Trial (DCCT)/Epidemiology of Diabetes Interventions and Complications (EDIC) [46]. Further research to confirm this finding is necessary. 

#### Receptor for Advanced Glycation End Products Gene (RAGE)

Advanced glycation end products (AGEs) result from the non-enzymatic glycation of proteins and lipids caused by hyperglycemia. It has been suggested that AGEs accumulation contributes to diabetic complications through direct tissue damage, as well as through activating specific receptors for AGE (RAGE)[47-49]. The role of RAGE (a member of the immunoglobulin superfamily located in chromosome 6p21.3) in the pathophysiology of DR, makes its polymorphisms good candidates for influencing DR development. 

The -374A T/A polymorphism influences a transcription factor-binding site which, in turn, leads to the upregulation of RAGE transcription. Some studies have found an association between the -374 T/A polymorphism and sight-threatening DR [50, 51]. However, these findings have not been supported by other studies [52-56]. 

Gly82Ser and -429 T/C polymorphisms may also increase the risk of DR in some populations [56-58], although this relationship has not been significant in other studies [51, 52, 54, 55, 59]. 

Two meta-analysis performed in Caucasian and Asian populations [60, 61], did not found a significant association between these polymorphisms (-374A, Gly82Ser and -429 T/C) and DR. Interestingly, the meta-analysis in which East Asian subjects were the predominant population, the 1704T allele was associated with an increased risk of DR [61]. Moreover, recently, it has been reported in a Malaysian population that the 2245A allele is associated with the development of DR [62]. Further studies are necessary to corroborate these findings.

#### Endothelial Nitric Oxide Synthase (eNOS)

eNOS catalyses the formation of the molecule NO from the substrate L-arginine [63], and has been implicated in the pathogenesis of diabetic vascular complications. For instance, diabetic eNOS(-/-) mice exhibit a significantly wider range of advanced retinal vascular complications [64]. The eNOS gene (*NOS*) is located in chromosome 7q35-26, and its polymorphisms have been related to an increase in the risk of developing DR [28]. 

The a/b polymorphism of the intron 4 eNOS gene was found to have a significant association with DR in a West African cohort [65]. However, more recent studies, including a meta-analysis, have not found a significant association between this polymorphism and the early onset of mycroangiopathy [66, 67]. Similarly, the C allele of T-786C polymorphism was initially associated with DR [68] but this has not been confirmed by other studies [28, 65, 67]. 

Recently, Zhao *et al.* [69] conducted a meta-analysis in which the association of those aforementioned polymorphisms was analyzed. A significant association was found between the intron 4a allele of the 4b/a polymorphism and a reduced risk of DR. The C allele of T-786C polymorphism may be a protective factor for PDR. In addition, they could not prove an association between the G894T polymorphism and DR.

#### Angiotensin-I Converting Enzyme (ACE)

The *ACE* gene, which is located in 17q23 chromosome [28], is one of the most studied candidate genes. ACE is part of the renin-angiotensin system and has been implicated in the development of DR. In fact, elevated ACE expression has been shown to have an adverse effect on retinal blood flow and vascular structure and it also promotes the formation of new retinal blood vessels [70].

The insertion/deletion (I/D) polymorphism has been one of the most investigated and has been associated with DR [71, 72]. A recent meta-analyses in which more than two thousand Chinese patients were included showed that the I/D polymorphism was associated with PDR, but not with non-proliferative DR [73].

Finally, the ACE 2350 G/A polymorphism, which among the polymorphisms of this gene is known to have the most significant effect on plasma ACE concentrations, has been associated with DR in Han Chinese patients with type 2 diabetes [74]. 

#### Others

The erythropoietin (EPO) gene is located on 7q21 and encodes an angiogenic factor expressed in the retina and the kidney. There is a well documented association between PDR and diabetic nephropathy, and this could be explained by the existence of common genetic factors. Tong *et al.* [75] genotyped 19 SNPs from 11 candidate genes in diabetic patients with or without PDR and end-stage renal disease and tested for allelic associations. This study showed an association between a functional EPO promoter polymorphism (rs1617640) and both eye and kidney diabetic microvascular complications in a European-American population. The risk allele (T) was associated with elevated EPO levels in the vitreous fluid [75]. These results were confirmed in a large case-control meta-analysis of diabetic nephropathy in type 1 diabetic patients. However, no association was observed between the rs1617640 polymorphism and PDR when this microvascular complication was analyzed irrespective the renal failure [76]. It should be noted that EPO could play a neuroprotective role in the early stages of DR and it protects the retinal pigment epithelium against the increase of permeability induced by diabetic conditions [77-79]. However, EPO acts synergistically with VEGF in advanced stages of DR, thus favouring angiogensis and PDR [80]. Therefore, further studies to clarify this double-edged sword role of EPO in the pathogenesis of DR are needed. 

On the other hand, specific genetic loci that are associated with differences in HbA1c between people with type 1 diabetes have been convincingly identified, and they have also been shown to be associated with diabetic complications [81]. 

Finally, a recent meta-analysis provides evidence that the Pro12Ala polymorphism of the peroxisome proliferator–activated receptor-γ2 (PPARγ2) is associated with DR. The Ala allele of the Pro12Ala polymorphism has a protective effect on the incidence of DR in Caucasian but not in Asian type 2 diabetic patients [82]. 

### Linkage Studies

Linkage studies analyze shared alleles among family members under the assumption that these predispose to a more aggressive development of DR. One of the earliest linkage studies was performed by studying the genome of identical twins with DR, and a concordance of 68% in type 1 diabetes and 95% in type 2 diabetes was found [20], but in this study no specific genome regions were identified. Almost twenty years later a linkage analysis among Pima Indians with type 2 diabetes was performed to identify susceptibility genes for both DR and nephropathy [83]. Two regions, one on chromosome 3 and one on chromosome 9 indicated a tentative linkage with retinopathy, although none of them was significant. It should be noted that a tentative linkage between a region relatively close to the angiotensine II receptor gene (*AGTR1*), which is located on chromosome 3q2125, and both nephropathy and retinopathy was found in this study [83]. 

More recently, a genome-wide linkage analysis designed to identify loci for DR, taking into account the severity of the DR was also performed in a Pima Indian population. A modest level of familial aggregation and suggestive evidence for linkage to chromosome 1p36 was found [84]. The genes located in this region were: peptidyl arginine deiminases (PADI) 1, 2, 3, 4 and 6, CASP-9, CLCN-Ka and CLCN-Kb, and none of these genes had previously been associated with DR. The main weakness of this study was that only 4% of the cohort had moderate non-PDR or worse. This means that their observations were limited to relatively early stages of retinopathy and so may not have described genetic factors associated with more severe disease. Further linkage studies or candidate gene studies (with PADI, CASP-9, CLCN-Ka and CLCN-Kb) would be useful to confirm this association. The difference between these two studies into a Pima Indian population could be explained by the sample size and because diabetes duration and severity of DR were not considered in the first study. 

Another linkage study was performed into a population of Mexican Americans but it did not reach the level of genome-wide significance for any gene [85]. The strongest evidence involved the proximal end of chromosome 12. Several candidate genes potentially involved with DR occurred in this region such as WNT5B (associated with adipogenesis and type 2 diabetes), TULP3 (associated with retinal degeneration), GNB3, WNK1, SCNN1A (the last three associated with hypertension), ING4 (associated with angiogenesis) and OLR1 (associated with hypertensive vascular damage and involved in choroidal neovascularization in age-related macular degeneration). The next strongest evidence, although without statistical significance occurred in chromosome 3 in which are located some genes related with retinal diseases such as ROBO2 (associated with retinal development), PROS1 (associated with venous thrombosis and retinopathy of prematurity), ARL6 (associated with Bardet-Bield Syndrome with retinal dystrophy) and IMPG2 (associated with retinal interphotoreceptor matrix proteoglycan) [85]. 

### Genome-Wide Association Studies

Genome-Wide Association Studies (GWAS) involve the massive evaluation of SNPs in large samples, thus representing a new tool for identifying candidate genes. This is based on the genome-wide characterization of linkage disequilibrium, the phenomenon by which adjacent polymorphisms are correlated with each other because of their co-segregation from one generation to the next. Using these data, publicly available from the HapMap database, a set of SNPs can be selected that most efficiently capture the 10 million or so common polymorphisms in the human genome. In addition, technology based on microarrays is used and several hundred thousand selected SNPs can be typed in a single assay [86]. We will review the first steps in the genetics of DR with this unique approach.

The genome-wide meta-analysis association data for severe DR in two large cohorts of type 1 diabetes (the Genetics of Kidney in Diabetes and the Epidemiology of Diabetes Intervention and Control Trial studies) did not find any significant genome-wide association. However, a relationship between the intergenic SNP rs476141 and the presence of severe DR was found. The intergenic SNP rs476141 is located in a region between two genes, AKT3 and ZNF238. Interestingly, AKT3 is a serine/threonine kinase regulating cell survival, insulin signaling and angiogenesis, and has been shown to be activated by platelet-derived growth factor and insulin-like growth factor 1, which have both been implicated in PDR [87]. This association was not replicated in a cohort of type 1 diabetic patients from the Wisconsin Epidemiologic Study of Diabetic Retinopathy but new genetic loci potentially associated with severe DR were identified [88]. The strongest association was at rs4865047, an intronic SNP in the gene *CEP125*. This gene is expressed in the retina and is related to TGF-β signaling. However, further studies to confirm these associations are needed.

The Candidate-gene Association Resource (CARe) was a collaborative study aimed at investigating the association analyses between genotypes and cardiovascular disease phenotypes [89]. It comprised >40,000 participants from nine cohorts who had been genotyped for 49,320 SNPs from approximately 2,000 candidate genes postulated or known to increase the risk of cardiovascular, metabolic, and inflammatory diseases [90]. It included 2691 type 2 diabetic subjects with fundus photographs of multiple ethnicities. Therefore, the CARe framework provided an opportunity to investigate genetic associations for DR with a candidate gene approach. In this regard it has recently been investigated whether variants in cardiovascular candidate genes, some of which have been previously associated with type 2 diabetes, DR, and diabetic nephropathy, are associated with DR in the CARe [91]. In this study three SNPs in the P-selectin (SELP) gene were associated with DR in the European American population but they were unable to replicate this finding in other ethnic groups. In this regard, it has been found that genetic effects identified by GWAS are not consistent across populations of different ancestry [92]. Therefore, genomic risk markers need a separate evaluation in different ethnic groups.

GWAS studies have some limitations. The commercial arrays used for GWA scans are designed to provide excellent coverage of common SNPs, but have only limited potential to capture rare and low frequency variants (i.e. those with a minor allele frequency below 5% [93]. Furthermore, if susceptibility alleles have minor allele frequencies of less than 0.1 and their effect sizes are less than an odds ratio of 1.3, then unrealistically large sample sizes of more than 10,000 cases and 10,000 controls (or 10,000 families) would be required to achieve statistical support for a disease association [94].

Mild retinopathy, defined as the presence of isolated microaneurysms or dot-blot hemorrhages, is observed in 5 to 15% of the general non-diabetic population [95]. Jensen *et al.* [96] conducted a GWAS to examine the association between 2.5 million SNPs and the presence of isolated microaneurysms or dot-blot hemorrhages in a non diabetic population. The results from this study showed little evidence that the presence of mild retinopathy in individuals without diabetes was associated with SNPs. However, further studies are needed to explore the remaining highly suggestive SNPs such as rs12155400 (located in the histone deacetylase 9 gene [*HDAC9*] on chromosome 9 7). This may include fine mapping in *HDAC9*, gene-environment interaction studies or pathway analyses. 

## CONCLUDING REMARKS

There have been extensive research efforts to understand the genetics of DR. The genetic associations of DR are useful as a research tool but may also have a relevant clinical value in identifying patients at a high risk of developing RD. Thus, more frequent examinations of high-risk patients, and less frequent examinations of low-risk patients, may lead to the more efficient targeting of health care resources. 

Heritability has been estimated to be as high as 27% for DR and 52% for PDR. Efforts to unravel the human genetics of DR have been undertaken using the candidate gene, linkage approaches, and more recently GWAS. A large number of putative genes and genetic variants have been reported in the literature and some of them exhibit consistent associations with DR (ALR2, VEGF and RAGE genes). However, these results have not been replicated in multiple populations and, therefore, no genes have achieved widespread acceptance as conferring a high risk of DR. These inconsistencies may reflect the lack of an accurate definition of DR, the small subject numbers, underlying genetic differences between study populations, and differences in study design. In addition the duration of diabetes is a key factor which should be considered in the analysis of the results. Finally, the overlap of DR with ‘non-diabetic’ retinopathy, which has been found to occur in 5-15% of subjects without clinical diabetes, could also be a confounding factor. It is expected that GWAS will provide novel insights into genetic susceptibility to DR in the near future. Moreover, the genomic sequence data to be published in the near future will be useful to define genetic factors that confer a risk for the development and progression of DR.

In summary, a genetic approach in order to identify new candidates in the pathogenesis of DR would permit us to design more targeted therapeutic strategies in order to decrease this devastating complication of diabetes. Basic researchers, ophthalmologists, diabetologists, and geneticists should work together in order to gain new insights into this issue. 

## Figures and Tables

**Table 1.** Main Studies on Candidate Genes Related to Diabetic Retinopathy

Genes	Polymorphisms		OR	p	n	Population	Type of Study	thM	Special Features	Reference
*AKR1B1*	rs759853 T allele		0.49	0.0001	2207	-	Meta-analysis	1 and 2	Protection against DR in type 1 diabetes.	[27]
	(CA)n microsatellite	z-2 allele	2.33	0.0002	1973	-	Meta-analysis	1 and 2	Weaker association in type 1 diabetes than type 2 diabetes. Associated with younger onset of diabetes.	[27]
		z+2 allele	0.58	0.02	1693	-	Meta-analysis	1 and 2	Protective effect. Associated with younger onset on diabetes.	[27]
	rs9640883		1.62	0.0005	514	Australian	Case-control	1 and 2		[25]
*VEGF*	+405	CC genotype	3.2	0.004	452	Japanese	Case-control	2	VEGF serum levels are higher in healthy subjects with the CC genotype.	[33]
			-	NS	919	Caucasian	Case-control	2	No statistical association with DN.	[34]
		GG genotype	1.87	0.039	398	Iranian	Case-control	2	The polymorphism increases the risk of PDR vs NPDR.	[35]
			-	NS	919	Caucasian	Case-control	2	No statistical association with DN.	[34]
		CG genotype	2.3	0.008	210	Indian	Case-control	2	Case group: patients with PDR.	[40]
			1.66	0.42	213	South Indian	Case-control	2	The distribution of this genotype was significantly higher in patients with DR and microalbuminuria than in those patients with microalbuminuria but without DR.	[43]
		G allele	0.62	0.0005	4226	-	Meta-analysis	2	Significant association in patients with NPDR but not in those patients with PDR.	[27]
		All genotypes	-	NS	469	Japanese	Case-control	2		[44]
	-2578	AA genotype	7.5 (Rm)	0.002	469	Japanese	Case-control	2	Results from the duration weighed sub-analysis.	[44]
		CA genotype	-	0.002	40	Caucasian	Case-control	2	CC genotype may be associated with susceptibility to diabetic PAD while the CA genotype may be associated with susceptibility to DR.	[36]
	SRp55 2994		2.1	0.030	258	Caucasian	Case-control	1 and 2	Associated with PDR.	[45]
*RAGE*	-374A T/A		1.25 (T1DM) 1.16 (T2DM)	NS NS	2737	Scandinavian	Case-control	1 and 2	The frequency of the A allele was higher in patients with sight-threatening DR.	[50]
			1.814	0.048	528	South India	Case-control	2	Modest association with NPDR.	[51]
			-	NS	577	Malaysian	Case-control	2		[52]
			-	NS	703	Caucasian, African-Brazilians	Case-control	2		[53]
			-	NS	669	Chinese	Case-control	2		[54]
			0.76	NS	704	Indian	Case-control	2	Significant association between SNP rs2070600 of RAGE gene and DR.	[56]
			0.96	NS	15194	Most East Asian and Caucasian	Meta-analysis	1 and 2		[61]
	Gly82Ser		2.964	0.029	688	Chinese	Case-control	2		[57]
			-	NS	210	Indian	Case-control	2		[59]
			-	NS	3049	Asian	Meta-analysis	2		[60]
			0.95	NS	12296	Most East Asian and Caucasian	Meta-analysis	1 and 2		[61]
	-429 T/C		-	NS	1917	Asian and Caucasian.	Meta-analysis	2		[60]
			1.02	NS	9550	Most East Asian and Caucasian	Meta-analysis	1 and 2		[61]
	2245G/A. A allele		3.06	<0.001	577	Malaysian	Case-control	2	Both NPDR and PDR.	[62]
	1704G/T. T allele		-	NS	1426	Asian and Caucasian	Meta-analysis	2		[60]
			1.09	NS	6145	Most East Asian and Caucasians	Meta-analysis	1 and 2	Subgroup analysis by ethnicity indicated that allele 1704T conferred a signiﬁcant increase of risk in East Asians but not in Caucasians.	[61]
*NOS3 *	4 a/b polymorphism bb genotype		2.4	0.013	575	West African	Case-control	2		[65]
			-	NS	630	Caucasian-Brazilian	Case-control	2		[67]
			-	NS	3377	-	Meta-analysis	1 and 2		[27]
			0.846	0.015	3147	Most Asian and Caucasian	Meta-analysis	Most 2	Protective effect. No statistical association was found in the dominant, recessive or co-dominant models. No statistical was found after the stratification for ethnicity.	[69]
	C allele of T786C		3.4	<0.001	249	Caucasian	Case-control	1	They also found a significant association with diabetes.	[68]
			-	NS	630	Caucasian-Brazilian	Case-control	2		[67]
			-	NS	2147	Most Caucasian	Meta-analysis	Most 2		[69]
	G894T		-	NS	2819	Most Caucasian and Asian	Meta-analysis	2		[69]
*ACE*	I/D polymorphism	ID genotype	1.831	0.026	384	Iranian	Case-control	2		[71]
		DD genotype	3.516	0.015	136	Iranian	Case-control	2	PDR vs. NPDR.	[72]
			DD vs. II: 1.73	<0.05	2224	Chinese	Met-analysis	Not specified	Subgroup analyses based on the type of DR showed that the effect was significant for PDR but not NPDR.	[73]
			DD+ID vs. II: 1.41	<0.05						
			DD vs. ID+II: 1.55	<0.05						
	2350 G/A AA genotype		4.4887	0.0006	235	Han Chinese	Case-control	2		[74]

## ABBREVIATIONS

ACE: = Angiotensin-I Converting Enzyme (ACE)
AGEs: = Advanced glycation end products
ALR2: = Aldose reductase
DR: = Diabetic retinopathy
GWAS: = Genome-wide association studies
I/D: = Insertion/deletion
PADI: = Peptidyl arginin deaminase
PPARγ2: = Peroxisome proliferator-activated receptor-γ2
RAGE: = Receptor for advanced glycation end products
SNP: = Single nucleotide polymorphism
VEGF: = Vascular endothelial growth factor
